# Transactions of the College of Physicians of Philadelphia, from Sept. 6th, 1848, to Jan. 2d 1849

**Published:** 1849

**Authors:** 


### ARTICLE IV.

*Transactions of the College of Physicians of Philadelphia, from Sept. 6th,* 1848, *to Jan. 2d* 1849.

The appearance of these reports has become a matter of much interest, as they from time to time give a synopsis of the results of practice, by a large and highly intelligent body of physicians.

The present number is highly interesting, and the annual reports of Dr. Coats on the theory and practice of medicine, and of Dr. Griscon on Midwifery, are valuable contributions to our medical literature. E

